# Phenotypic, functional and metabolic features of B cells from a small cohort of breast cancer patients

**DOI:** 10.3389/fonc.2026.1944615

**Published:** 2026-09-25

**Authors:** Kate Miller, Andrew Gelsomini, Maria Romero, Denisse Garcia, Alain Diaz, Valquiria Bueno, Jenna Hansen, Roland Jurecic, Michael H. Antoni, Daniela Frasca

**Affiliations:** 1 Department of Microbiology and Immunology, University of Miami Miller School of Medicine, Miami, FL, United States; 2 Department of Microbiology, Immunology and Parasitology, Federal University of São Paulo, São Paulo, Brazil; 3 Department of Psychology, University of Miami, Coral Gables, FL, United States; 4 Sylvester Comprehensive Cancer Center, University of Miami Miller School of Medicine, Miami, FL, United States; 5 Department of Psychiatry and Behavioral Sciences, University of Miami Miller School of Medicine, Miami, FL, United States

**Keywords:** B cells, breast cancer, inflammation, lactate, metabolism

## Abstract

**Objective:**

Identify and characterize breast cancer (BC)-induced changes of humoral immunity and B cell function in a small cohort of treatment-naïve breast cancer patients (BCPs) as compared to healthy controls (HCs).

**Methods:**

The peripheral blood from newly diagnosed, treatment-naive breast cancer patients (BCPs) and age-matched cancer-free individuals (HCs) was collected. B cells were isolated and cellular, molecular, functional and metabolic analyses were performed.

**Results:**

There is a redistribution of B cell subsets in the blood of BCPs versus HCs with increased frequencies of pro-inflammatory B cell subsets in BCPs. B cells from BCPs secrete higher amounts of pro-inflammatory cytokines than B cells from HCs, due to the activation of the transcription factor NF-kB. B cells from BCPs also secrete high amounts of the metabolite lactate and are highly glycolytic, as shown in a Seahorse mitostress test. This hyper-metabolic phenotype can be inhibited at least *in vitro* by blocking lactate transport.

**Conclusion:**

Results have identified a set of distinct and significant changes in the circulating B cells of our cohort of BCPs that may help to establish a baseline B cell signature of BCPs before therapy, to predict B cell performance during therapy by identifying immunosuppressive cells, and to design precision therapeutic approaches.

## 1 Introduction

Breast cancer (BC) is the most frequently diagnosed malignancy in women worldwide (1). In 2025, an estimate of >300,000 new cases of invasive BC have been diagnosed in the U.S., making it the most common non-skin cancer accounting for almost 30% of all new cancer diagnoses in females (https://www.nationalbreastcancer.org/breast-cancer-facts/).

Our ongoing studies and those by several other groups have revealed that BC starts to adversely change the patient’s immune system from early on in its development and in the timeframe before the start of cancer therapy (2–4). BC causes profound cellular, molecular and metabolic changes that lead to functional impairments of the immune system in treatment-naive hosts, which in turn negatively affect not only immune responses but can also impact the efficacy of treatment as well as clinical, psychological, and physiological outcomes. Therefore, characterizing BC-related changes in the immune system of treatment-naive BC patients (BCPs) is needed to find ways to improve the immune health of BCPs and to identify the cellular and molecular mechanisms involved.

In recent years, B cells have become an increasingly important focus in BC research due to their capacity to regulate cancer progression and response to therapeutic interventions. However, B cells may be a double-edged sword in BC, as they can promote anti-tumor immunity through anti-tumor antibody secretion, antigen presentation or activation of anti-tumor T cells, or alternatively they can contribute to tumor progression (5). Whether B cells exert a positive or negative response to cancer cells is largely impacted by the distribution of the four major subsets of blood B cells, naive, IgM memory, switched memory, and double negative (DN) memory cells. DN B cells are the most pro-inflammatory B cells (6) and are characterized by the expression of RNA for multiple markers of the senescence-associated secretory phenotype, SASP (7, 8) which has been associated with worse recurrence-free survival and overall survival in cancer patients (9). As such, an increase in the frequency of DN B cells may promote poorer responses to treatment. Therefore, B cell modulation in BCPs offers promising avenues for precision therapeutic approaches, in combination with either chemotherapy or B cell-specific immune checkpoints.

In the present study, we have identified and characterized BC-induced cellular, molecular, metabolic and functional changes in humoral immunity and B cell function of a small cohort of treatment-naïve BCPs versus healthy controls (HCs). Results herein show a redistribution of B cell subsets in the blood of BCPs versus HCs, characterized by a decrease in the frequency of naïve B cells and an increase in memory B cell frequencies, with the subset of DN B cells showing the highest level of expansion as compared to the other memory B cell subsets. B cells from BCPs secrete higher amounts of pro-inflammatory cytokines than B cells from HCs, which is associated with the activation of the transcription factor NF-kB, and they also secrete high amounts of the metabolite lactate. We show that this hyper-glycolytic phenotype can be inhibited at least *in vitro* by blocking lactate transport.

## 2 Materials and methods

### 2.1 Participants

BC patients (BCPs, n=20) and Healthy Controls (HCs, n=20) were recruited at the University of Miami Miller School of Medicine (UM-MSOM). The study was reviewed and approved by UM-MSOM Institutional Review Board [IRB, protocols #20180366 (Jurecic PI), #20160525 (Antoni PI), #20160542 (Frasca PI)], which reviews all human research conducted under the auspices of the University of Miami. All participants signed an informed consent.

BCPs were newly diagnosed with Invasive Ductal Carcinoma [hormone receptor (HR)+, HER2-, which represent the most common phenotype], stage I-III, grade 2-3, Ki-67 60-67%. All BCPs were treatment-naïve. BCPs and HCs were age matched, all in menopause for at least 2 years before recruitment.

The characteristics of the recruited cohorts of BCPs and HCs are shown in **Table 1**.

**Table 1:** Characteristics of enrolled participants.

	BCPs	HCs
Age, mean±SE	58±1	57±1
BMI*, mean±SE	27±1	27±2
Race	White	White
Ethnicity (Caucasian/Hispanic)	10/10	9/11

*BMI, Body Mass Index (kg/m^2^)

Exclusion criteria for both BCPs and HCs were: 1) a diagnosis of cancer in the 2-year period before recruitment (with the exception of non-melanoma skin cancer), 2) a diagnosis of major mental disorders, 3) a co-morbid medical condition with known effects on the immune system (HIV infection or autoimmune diseases), 4) current medications known to affect the immune system, 5) smoking.

### 2.2 PBMC collection

Experiments were performed using cells isolated from the peripheral blood of BCPs and HCs. Peripheral Blood Mononuclear cells (PBMC) were isolated from blood drawn in Vacutainer CPT tubes (BD 362761). After blood was drawn, tubes were gently mixed and centrifuged at room temperature for 30 minutes at 2,800 rpm. PBMC are in the white cell layer just below the plasma layer. PBMC were cryopreserved in freezing medium [DMSO (10%) + Fetal Bovine Serum (90%)], and kept at -155° C until use. On the day of the experiment, PBMC were thawed and cell viability was evaluated by trypan blue counting. PBMC were discarded if viability was <75%. Viability was further confirmed by flow cytometry experiments, performed in all participants (see below).

### 2.3 B cell isolation and stimulation

Total B cells were isolated from PBMC using magnetic CD19 Microbeads (Miltenyi 130-050-301), following the MiniMACS protocol (20 µL Microbeads + 80 µL PBS, for 10^7^ cells). Cell preparations were usually >95% pure. After enrichment, B cells were maintained in complete medium, c-RPMI [RPMI 1640 (Gibco ThermoFisher 11875-093), supplemented with 10% FBS (Gibco 10437-028), 100 U/mL Penicillin-Streptomycin (Gibco 15140-122), and 2 mM L-glutamine Gibco 25030-081)].

For RNA isolation and quantitative (q)PCR, B cells were resuspended in TRIzol (Ambion) at the concentration of 10^6^ cells/500 µl.

For cytokine and lactate secretion in culture supernatants, B cells at the concentration of 10^6^/mL were stimulated for 48 hrs with 1 µg/10^6^ cells of CpG (ODN 2006 InVivogen). Cytokines were measured by a Cytometric Bead Array (CBA), whereas lactate was measured by a colorimetric assay.

In some experiments, B cells were left untreated or they were pre-treated overnight with the small molecule FX11 [3-dihydroxy-6-methyl-7-(phenylmethyl)-4-propylnaphthalene-1- carboxylic acid] that inhibits the enzyme lactate dehydrogenase A, LDHA (EMD Millipore 427218), or with a polyclonal antibody blocking the lactate transporter SLC5A12 (ThermoFisher Invitrogen PA5-110389).

### 2.4 Flow cytometry

PBMC (10^6^/mL) were membrane stained for 20 min at room temperature with a LIVE/DEAD™ Fixable Aqua Dead Cell Stain Kit (ThermoFisher L34966) and with the following fluorochrome-conjugated antibodies: anti-CD45 (Biolegend 304014, clone HI30), anti-CD19 (BD 555415, clone HIB19), anti-CD27 (BD 555441, clone M-T271) and anti-IgD (BD 555778, clone IA6-2) to measure the four major subsets of blood B cells, i.e. naive (IgD+CD27-), IgM memory (IgD+CD27+), switched memory (IgD-CD27+) and DN memory (IgD-CD27-) B cells. Moreover, B cells were analyzed for phenotypic markers of immune activation (IA) using the following fluorochrome-conjugated antibodies: anti-CD95 (Biolegend 305629, clone DX2), anti-CD11c (Biolegend 301637, clone 3.9), anti-PD-1 (Biolegend 329917, clone EH12.2H7), and anti-CXCR5 (Biolegend 356933, clone J252D4). Concentrations of the different fluorochrome-conjugated antibodies were standardized and validated in preliminary experiments. Staining was performed at room temperature for 20 minutes. In every experiment, 10^4^ to 10^5^ events in the B cell gate were acquired on a LSR-Fortessa (BD). Results were analyzed using FlowJo 10.10.1 software. Single color controls were included in every set of experiments for compensation. Isotype controls, as well as FMO (fluorescence minus one), were also used in every set of experiments to set up the gates.

PrimeFlow RNA assay, an amplified Fluorescence *In Situ* Hybridization in combination with flow cytometry, was used to measure nuclear content of the transcription factor NF-kB p65/RelA. Briefly, PBMC were membrane stained with Live/Dead kit, anti-CD45 and anti-CD19 antibodies. For RelA mRNA detection, target probe hybridization was performed using type 1 probe (AlexaFluor 647) for RelA (Affymetrix VA1-11996-PF). The negative control was the sample without the target probe. Cells were incubated for 2 hrs with the probe in a precisely calibrated incubator set at 40 °C. All samples were then incubated with the PreAmplification (PreAmp) reagent for 1.5 hrs and the Amplification (Amp) reagent for an additional 1.5 hrs at 40 °C. After signal amplification, cells were incubated with the label probe at 40 °C for 1 hr. Cells were washed and resuspended in staining buffer prior to acquisition. Approximately 10^5^ events were acquired from each sample on an LSR-Fortessa (BD) and analyzed using FlowJo 10.10.1 software. Compensation was completed using single color control samples. Isotype controls were also used in every experiment to set up the gates.

The CBA human TH1/TH2/TH17 kit (BD 560484) was used according to manufacturer’s instructions to measure the concentration of the following cytokines in culture supernatants: IL-2, IL-4, IL-6, IL-10, TNF-α, IFN-γ and IL-17A. Events (2x10^6^) were acquired at a LSR-Fortessa (BD), and results were analyzed using the FCAP Array™ Analysis Software.

### 2.5 RNA extraction, reverse transcriptase and quantitative (q)PCR

RNA was extracted from TRIzol according to the manufacturer’s protocol, eluted into 10 µl of 65 °C pre-heated elution buffer and stored at -80 °C until use.

RT reactions were performed in a Mastercycler Eppendorf Thermocycler to obtain cDNA. Briefly, 100 ng of total RNA were used as template for cDNA synthesis in the RT reaction. The qPCR reactions were conducted in MicroAmp 96-well plates and run in the ABI 7500 machine, each sample in triplicate. Calculations were made using the ABI software. For calculations, we determined the cycle number at which transcripts reached a significant threshold (Ct) for target genes and GAPDH (control). The difference in Ct values between GAPDH and the target gene was calculated as ΔCt. Then the relative amount of the target gene was expressed as 2^-ΔCt^ and indicated as qPCR values. All reagents were from ThermoFisher. Taqman primers were: GAPDH, Hs99999905_m1; Glut1/SLC2A1, Hs00892681; Glut2/SLC2A2, Hs01096908_m1; Glut3/SLC2A3, Hs00359840_m1; Glut4/SLC2A4, Hs00168966_m1; LDHA, Hs01378790_g1; PDHX, Hs00185790_m1.

### 2.6 Colorimetric assay to detect L-lactate

Culture supernatants were collected at day 2 of CpG stimulation to measure the secretion of lactate using the L-lactate assay kit (Cayman Chemicals 700510), following manufacturer’s instructions.

### 2.7 Seahorse mitostress test

The metabolic status of B cells was evaluated using the Seahorse mitostress test that allows the simultaneous measures of oxygen consumption rates (OCR) and extracellular acidification rates (ECAR), markers of oxidative phosphorylation (OXPHOS) and of overall cellular glycolysis, respectively. The mitostress test was performed in a Seahorse XFp extracellular flux analyzer (Agilent). B cells were initially incubated in Seahorse XF DMEM Medium (Agilent 103575), supplemented with 2 mM glutamine (Agilent 103579), 10 mM glucose (Agilent 103577) and 1 mM pyruvate (Agilent 103578), (200 μL of each reagent in 20 mL of medium). B cells, at the concentration of 2.5x10^5^/well, were seeded in a CellTAK (BD Biosciences)-coated plate in triplicate. B cells were sequentially treated with Oligomycin (1μM) that blocks ATP synthase, cellular energy production and proton flow, leading to a drop in OCR; then with FCCP (fluoro-carbonyl cyanide phenylhydrazone, 5 μM), an uncoupling agent that allows measures of maximal respiration; and finally with Rotenone/Antimycin (1 μM), that inhibit maximal respiratory capacity, showing that respiration is mitochondrial. Concentrations of the different drugs were standardized and validated in preliminary experiments.

Calculations were made as follows. In every experiment, we have first calculated the mean of the 3 measures of basal or maximal respiration over time for each well, and then the mean of the 3 wells. Spare respiratory capacity has been calculated by subtracting values of basal respiration from values of maximal respiration. Results of maximal respiration and spare respiratory capacity show biological replicates, shown in each figure as dots.

OCR results are not corrected for non-mitochondrial respiration, as unstimulated B cells show very low non-mitochondrial oxygen consumption.

### 2.8 Statistical analyses

To examine differences between 2 groups, unpaired Student’s t tests (two-tailed) were used. To examine differences between 6 groups, two-way repeated measures ANOVA was used. Group-wise differences were analyzed afterwards with Bonferroni’s multiple comparisons test, with p<.05 set as criterion for significance. Graphs and p values were calculated using GraphPad Prism version 10.1.0 software, which was also used to construct all graphs.

Multiple regression models that controlled for group were conducted using R programing language. Group was dummy coded (0=HPs and 1=BCPs). Regression models were run using the “stats” package.

tSNE visualization of the expression of IA markers on total B cells was done with FlowJo 10.10.1 software. Briefly, total B cells (gated on Live CD45+) from the same individual were first identified as a cluster, then heatmap overlay for each IA marker was independently generated, with each heatmap showing differential levels of expression across the total B cell pool. Individual analyses were run simultaneously.

## 3 Results

### 3.1 Redistribution of B cell subsets in the blood of BCPs versus HCs

We have characterized the composition of the circulating B cell pool of BCPs and HCs, using anti-IgD and anti-CD27 antibodies that allow the identification of the four major subsets of circulating B cells. Gating strategies are shown in Supplementary **Figure 1**.

**Figure 1:**
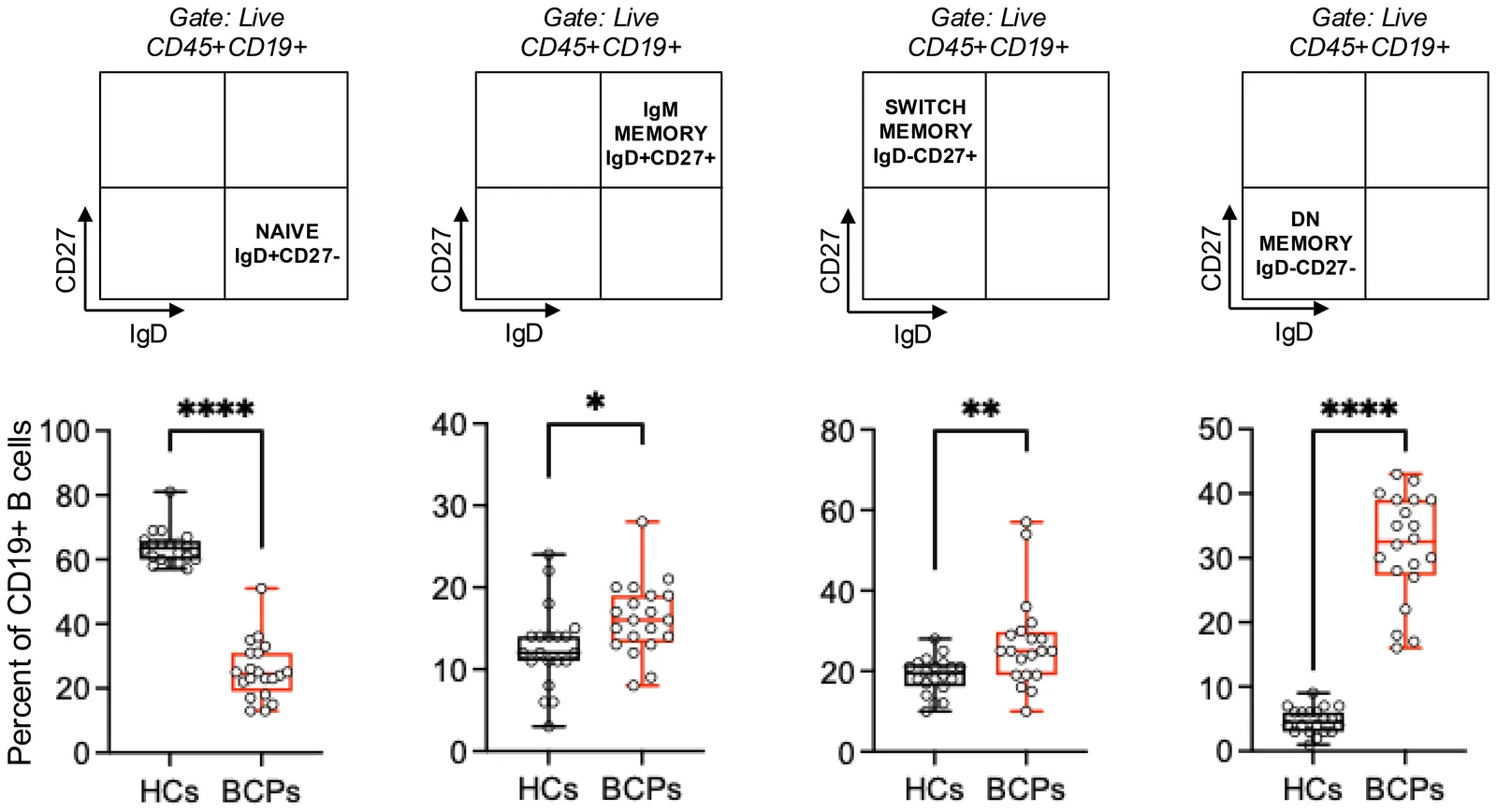
Redistribution of B cell subsets in the blood of BCPs versus HCs. PBMC from BCPs and HCs (2x10^6^/ml) were stained for 20 min at room temperature as indicated in Materials and Methods to identify the subsets of Naïve, IgM memory, Switch memory and DN memory B cells (top). Cumulative results from 20 BCPs and 20 HCs are shown (bottom). Results show mean frequencies±SE. Mean comparisons between groups were performed by unpaired Student’s t test (two-tailed). *p<0.05, **p<0.01, ****p<0.0001.

**Figure 1** (top) shows the major B cell subsets, gated on Live CD45+: naive (IgD+CD27-), IgM memory (IgD+CD27+), switched memory (swIg, IgD-CD27+) and DN memory (IgD-CD27-). Results in **Figure 1** (bottom) show that there are significant changes in the B cell pool of BCPs as compared to that of HCs. In particular, there is a decrease in the frequency of naïve B cells and an increase in the frequency of all memory B cell subsets in the blood of BCPs, with the subset of DN B cells showing the highest level of expansion as compared to the other memory B cell subsets. DN B cells are the most pro-inflammatory B cells that increase in the blood of individuals with inflammatory conditions and diseases, as we and several other groups have previously found (10–18). DN B cells express transcripts for multiple markers of the SASP (8, 19), most of which are markers of intrinsic inflammation and IA, whose expression is positively associated with immune cell dysfunction (20, 21). This inflammatory status of DN B cells is metabolically supported (6, 22). These results suggest that the higher frequencies of DN B cells in BCPs may be responsible for the higher inflammatory profile of the B cell pool in these patients (see also below).

### 3.2 Expression of IA markers on B cells from BCPs versus HCs

B cells from BCPs and HCs were also evaluated for the expression of the following markers of IA, known to be associated with B cell intrinsic inflammation and NF-kB activation, metabolic reprogramming, tissue recruitment and accumulation at sites of inflammation, pathogenic responses, and cell exhaustion: CD95 (23–25), CD11c (14, 26, 27), PD-1 (28–30) and CXCR5 (31, 32).

Results in **Figure 2A** show representative tSNE analyses from one HC (top) and one BCP (bottom) with the expression of all these IA markers being significantly higher in B cells from BCPs versus those from HCs, although to a different extent. Corresponding MFI expression of single markers in all HCs and BCPs is shown in **Figure 2B**. We also investigated co-expression of these IA markers in B cells from BCPs and HCs, and performed a combination analysis to measure frequencies of B cells expressing 1,2,3 or 4 of the above markers. Results in **Figure 2C** show higher frequencies of B cells co-expressing 2 or more IA markers in BCPs versus HCs. These results suggest higher levels of intrinsic inflammation in B cells from BCPs as compared to HCs.

**Figure 2:**
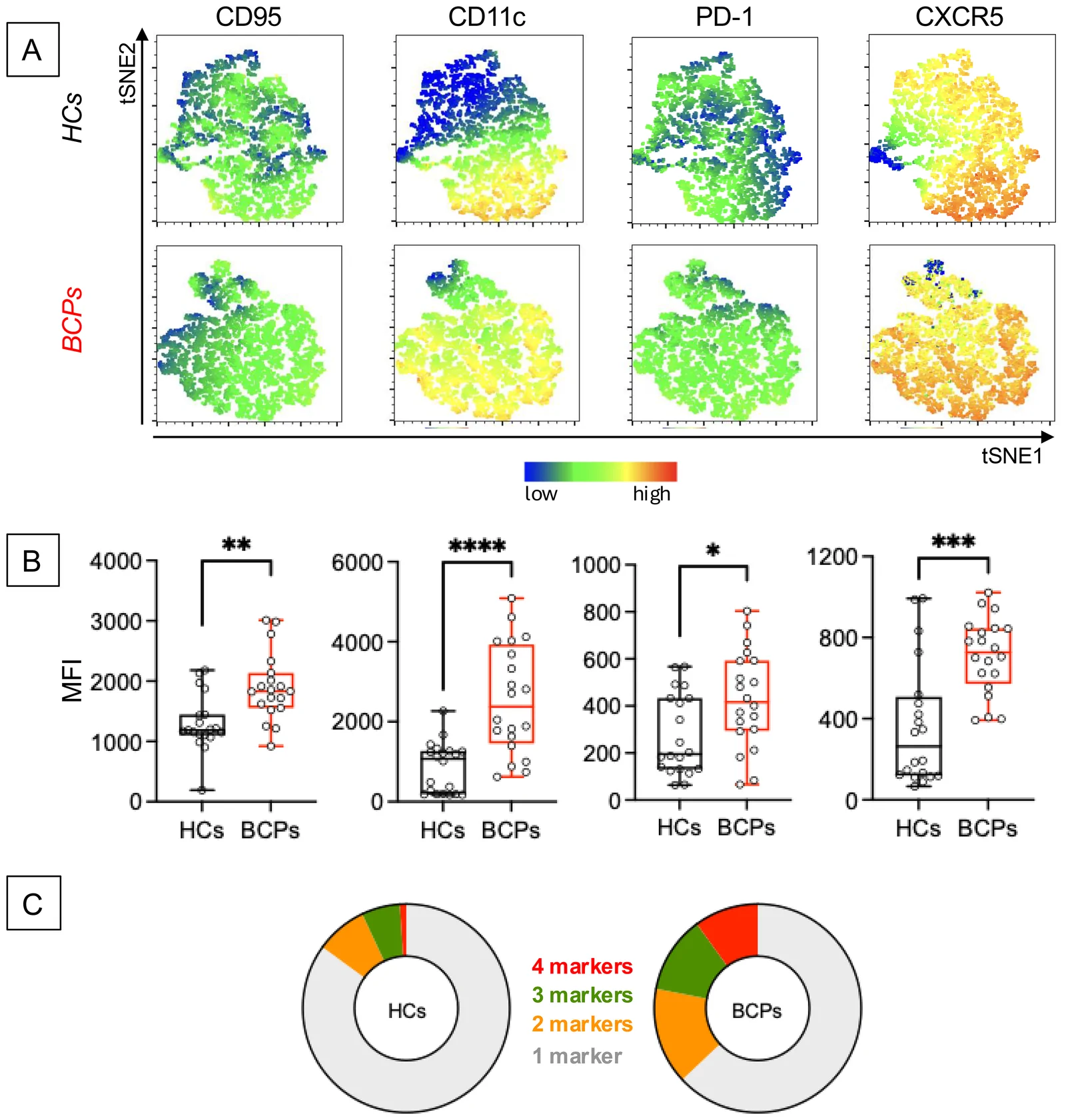
Expression of IA markers on B cells from BCPs versus HCs. PBMC from BCPs and HCs (2x10^6^/ml) were also stained to evaluate the expression of four markers of IA. **(A)** tSNE analyses of B cells (Live CD45+ CD19+) for the expression of the indicated IA markers, one representative individual/group. **(B)** Mean fluorescence intensity (MFI) for each marker. Results show mean MFI±SE from all individuals. **(C)** Combination gate analysis of four IA markers as shown by ring graphs. Each ring shows relative frequency of B cells expressing 1,2,3 or 4 markers simultaneously in each group. Mean comparisons between groups were performed by unpaired Student’s t test (two-tailed). *p<0.05, **p<0.01, ***p<0.001, ****p<0.0001.

### 3.3 B cells from BCPs are more metabolically active than those from HCs

Because the expression of IA markers is associated with intrinsic cell inflammation and IA, we next evaluated the metabolic status of B cells isolated from BCPs and HCs, as inflammatory B cells are metabolically supported, as we have previously shown (6, 22, 33). After enrichment from PBMC using CD19 magnetic beads, B cells were loaded on a mitostress test. B cells were left unstimulated because we have previously shown that the status of unstimulated B cells is associated with their capacity of being optimally stimulated either *in vivo* or *in vitro* (21).

Results in **Figure 3** show higher OCR (A), higher maximal respiration (B) and higher spare respiratory capacity (C) in B cells from BCPs versus those from HCs. The differences in ECAR were even higher and more significant than those in OCR (D), with higher maximal ECAR (E) and spare respiratory capacity (F), clearly showing that B cells from BCPs are able to effectively and quickly switch from OXPHOS to glycolysis after ATP synthase is blocked by the addition of oligomycin in the mitostress test.

**Figure 3:**
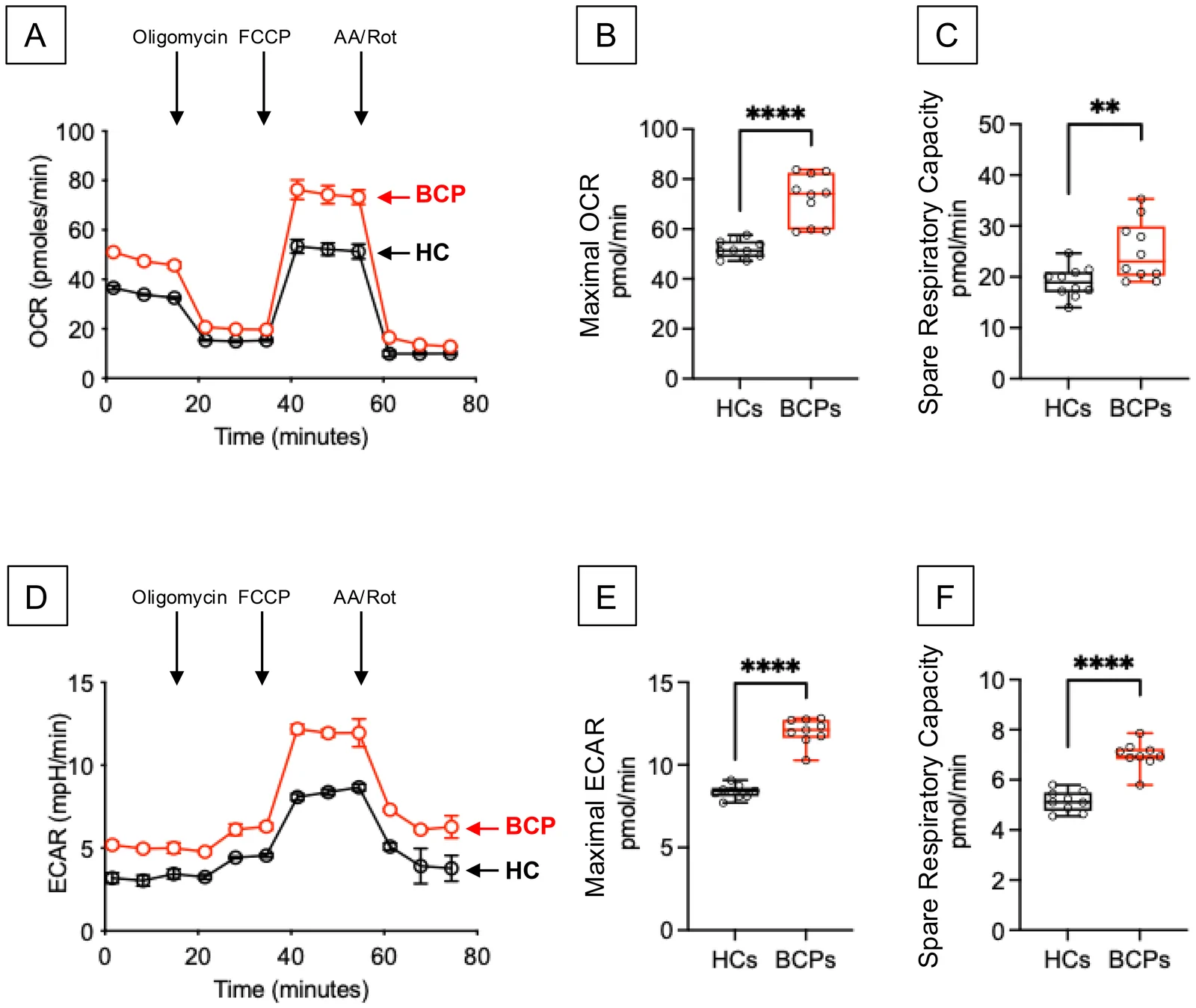
B cells from BCPs are more metabolic that those from HCs. B cells from BCPs and HCs were isolated from PBMC using magnetic beads and left unstimulated. B cells were seeded into the wells of an extracellular flux analyzer in triplicate (2.5x10^5^ cells/well) and run in a mitostress test. **(A)** OCR representative results from one HC and one BCP. **(B)** Maximal OCR respiratory capacity. **(C)** OCR spare respiratory capacity. **(D)** ECAR representative results from one HC and one BCP (same individuals in A). **(E)** Maximal ECAR respiratory capacity. **(F)** ECAR spare respiratory capacity. Spare respiratory capacity is calculated by subtracting basal values from maximal values. Results show mean of OCR/ECAR maximal respiration or spare respiratory capacity±SE Mean comparisons between groups were performed by unpaired Student’s t test (two-tailed). **p<0.01, ****p<0.0001.

### 3.4 B cells from BCPs express higher levels of transcripts for metabolic markers than those from HCs and secrete more lactate and pro-inflammatory cytokines upon stimulation

To confirm the above results, we measured by qPCR the expression of transcripts for enzymes involved in metabolic pathways associated with OCR and ECAR. We selected the transcripts for the two following enzymes. PDHX, a member of the pyruvate dehydrogenase (PDH) complex, that converts pyruvate into ATP, and LDHA, lactate dehydrogenase A, which converts pyruvate into lactate. The first enzyme is associated with measures of OXPHOS and OCR, whereas the second is associated with measures of glycolysis and ECAR. We also evaluated the expression of transcripts for glucose transporter 1 (Glut1), the major glucose transporter of human circulating B cells.

Results in **Figure 4** show higher expression of RNA for Glut1 (A), PDHX (B) and LDHA (C) in unstimulated B cells from BCPs as compared to those from HCs, with LDHA showing the largest difference of expression between the two groups. Conversely, transcripts for the other Gluts were comparable in B cells from the two groups (**Supplementary Figure 2**).

**Figure 4:**
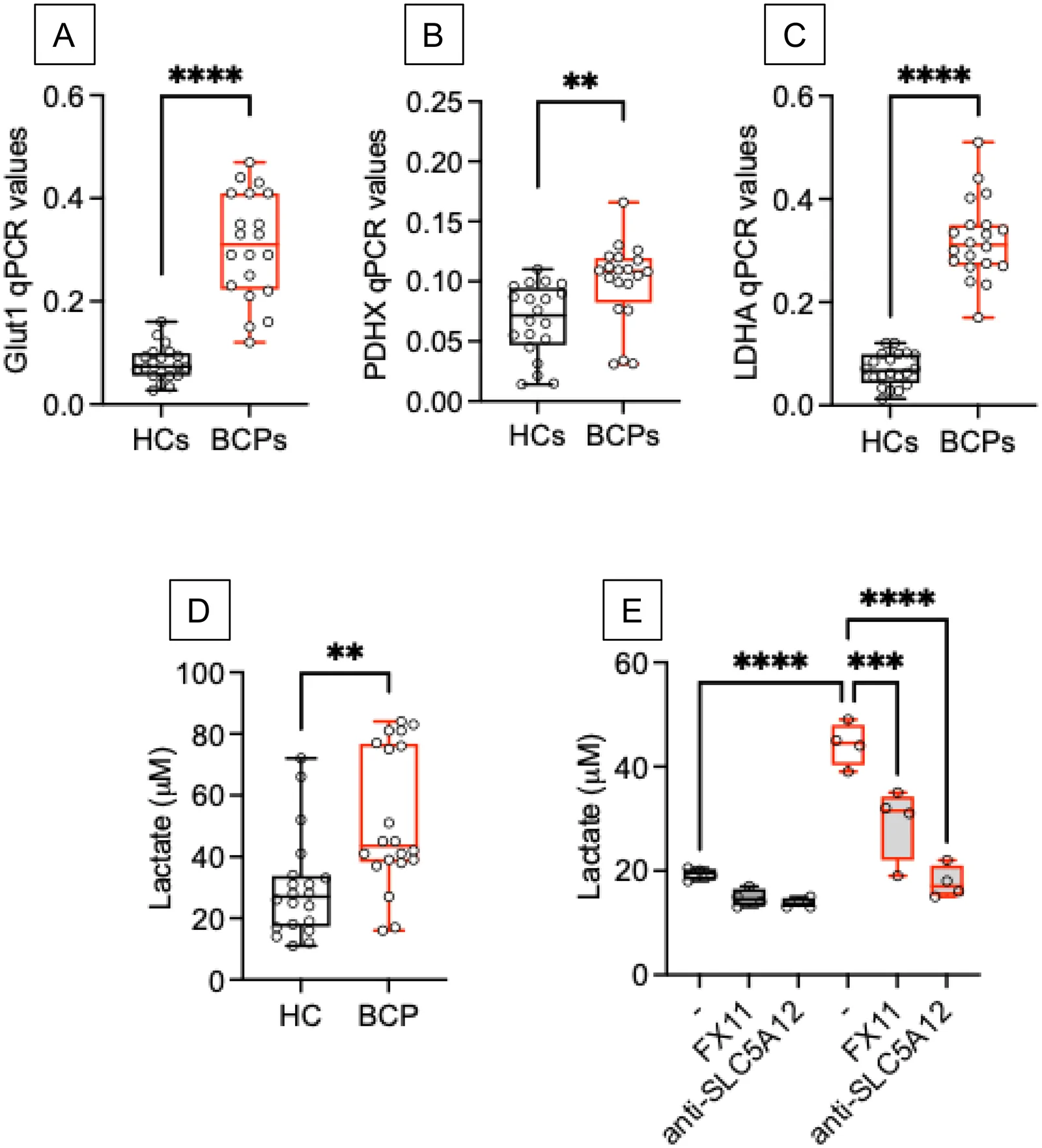
B cells from BCPs express higher levels of transcripts for metabolic markers than those from HCs and secrete more lactate upon stimulation. B cells from BCPs and HCs were isolated from PBMC using magnetic beads, left unstimulated and resuspended in TRIzol. Then RNA was isolated to evaluate the expression of transcripts for the metabolic markers Glut1 **(A)**, PDHX **(B)** and LDHA **(C)** by qPCR. Results are qPCR values (expressed as 2^-ΔCt^), mean±SE. **(D)** B cells were stimulated with CpG (1 µg/10^6^ cells) for 48 hrs, then lactate in culture supernatants was measured by a colorimetric assay. Results show mean of µM±SE. **(E)** B cells from BCPs and HCs (among those in **Figure 4C**) were left untreated or pre-treated overnight with FX11 (10 mM/10^6^ B cells), or with anti-SLC5A12 (1:500 dilution). Results show decrease in lactate secretion in culture supernatants, mean±SE. Mean comparisons between groups were performed by unpaired Student’s t test (two-tailed) **(A–D)**, and repeated measures two-way ANOVA **(E)**. **p<0.01, ***p<0.001, ****p<0.0001.

Because of this increase in LDHA, we then evaluated the amount of lactate secreted by CpG-stimulated B cells from BCPs and HCs. Results show that B cells from BCPs secrete more lactate than those from HCs (D).

We also showed that it is possible, at least *in vitro*, to inhibit lactate secretion in culture supernatants. To this purpose, we used two inhibitors: the small molecule FX11 that blocks the enzyme LDHA and the polyclonal antibody anti-SLC5A12 that inhibits the lactate transporter SLC5A12. This transporter acts as an electroneutral and low affinity sodium-dependent transporter that catalyzes the shuttling of many monocarboxylates, including lactate, through the plasma membrane, and can therefore be used for signaling purposes. Results in **Figure 4E** show that the pre-incubation of B cells with FX11 significantly reduced lactate secretion in culture supernatants of B cells from BCPs, and this inhibition was even more pronounced using the anti-SLC5A12 antibody. No inhibition was observed in B cells from HCs.

Results in **Figure 5** show that B cells from BCPs secreting higher amounts of lactate (**Figure 4D**) were also able to secrete higher amounts of the pro-inflammatory cytokines IL-6 (**Figure 5A**, left) and TNF-α (**Figure 5B**, left), upon *in vitro* stimulation. Moreover, the levels of these cytokines in CpG-stimulated B cells were positively associated with lactate secreted in the same culture supernatants (right). After regression analysis performed with group as a covariate, lactate was still positively associated with IL-6 secretion, whereas TNF-α was not, at least in these cohorts (**Table 2**). We have previously shown in other cohorts that lactate *in vitro* induces secretion of both IL-6 and TNF-α in CpG-stimulated B cells (22).

**Figure 5:**
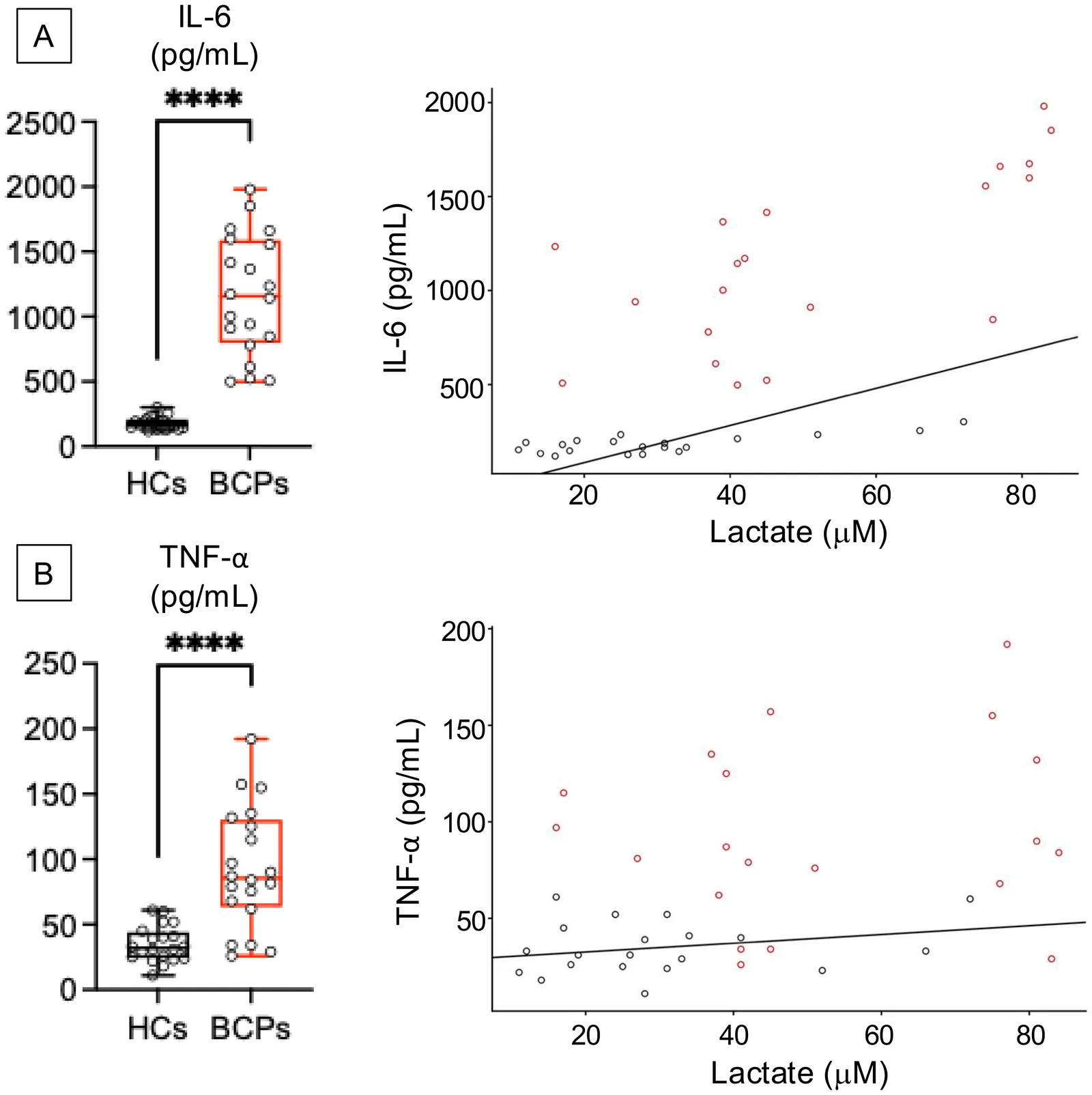
B cells from BCPs also secrete higher levels of pro-inflammatory cytokines upon stimulation. B cells were stimulated with CpG (1 µg/10^6^ cells) for 48 hrs, then pro-inflammatory cytokines in culture supernatants were measured by the CBA human TH1/TH2/TH17 kit. Left. Results show IL-6 **(A)** and TNF-α **(B)** secretion (means±SE). Mean comparisons between groups were performed by unpaired Student’s t test (two-tailed). ****p<0.0001. Right. Correlation of pro-inflammatory cytokine levels and lactate in CpG-stimulated B cell culture supernatants.

**Table 2:** Multiple regression models with lactate predicting pro-inflammatory cytokine secretion.

	*b*	*SE*	*p*
Model 1. Lactate as a Predictor of IL-6			
Lactate	**9.89**	**2.18**	**>.001**
	**765.08**	**97.06**	**>.001**
Model 2. Lactate as a Predictor of TNF-α			
Lactate	0.22	0.28	.42
**Group**	**53.13**	**12.41**	**>.001**

b: unstandardized regression coefficient.

SE: standard Error

Group was coded as 0 = HCs and 1=BCPs. Bold denotes statistically significant results.

### 3.5 B cells from BCPs express higher levels of transcripts for NF-kB than those from HCs

We then evaluated by PrimeFLOW RNA expression of *relA*, a member of the NF-kB family, in B cells from BCPs and HCs. This technique measures intracellular mRNA by an amplified fluorescence *in situ* hybridization technique in combination with flow cytometry and therefore allows the evaluation of *relA* nuclear expression in B cells. It is known that activation of the NF-κB transcription factor is associated with nuclear translocation of the p65/RelA component, and only nuclear p65/RelA binds the consensus sequence of target genes, including genes for pro-inflammatory cytokines (34). Flow cytometry panels in **Figure 6A** show the results from a representative experiment in which B cells from one BCP and one HC were evaluated. **Figure 6B** shows that B cells from BCPs express higher levels of transcripts for NF-kB than those from HCs, as expected from the results shown in **Figure 5**.

**Figure 6:**
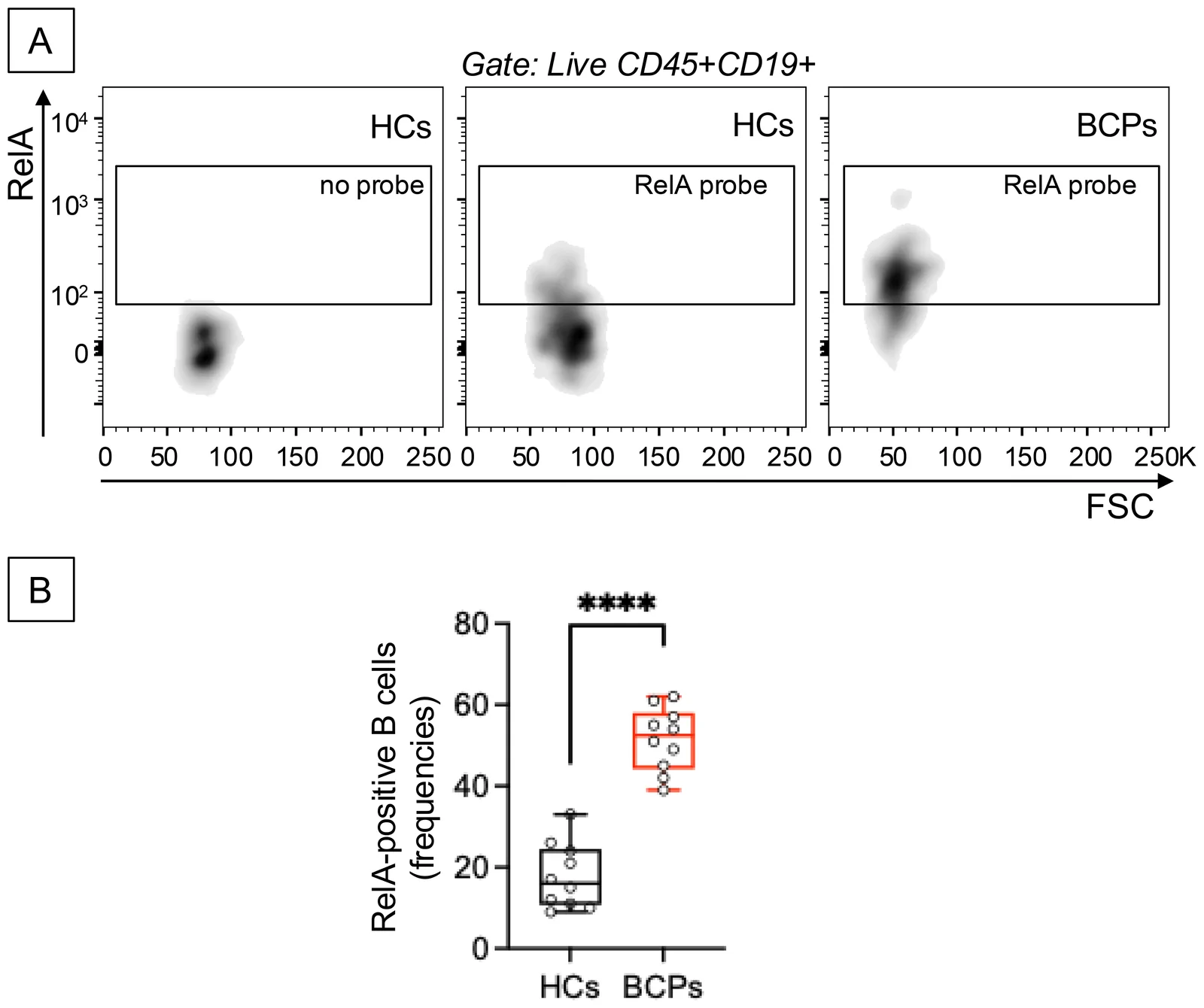
B cells from BCPs express higher levels of transcripts for NF-kB than those from HCs. PBMC were left unstimulated, then membrane stained with Live/Dead kit, anti-CD45 and anti-CD19 antibodies. **(A)** A representative experiment in which one HC and one BCP were evaluated. Negative control was the sample **(HC)** without the labeled probe. **(B)** Cumulative results from 10 BCPs and 10 HCs. Results show mean frequencies±SE. Mean comparisons between groups were performed by unpaired Student’s t test (two-tailed). ****p<0.0001.

## 4 Discussion and conclusions

The aim of our study was to find potential cellular biomarkers of blood-derived B cells that are predictive of BCPs’ immune status and that may be associated with the patients’ capacity to build up effective immune responses. It is known that BC can lead to compromised humoral immunity. In fact, BCPs, who represent vulnerable individuals, often succumb to infections (35) as they have poor responses to vaccination. This is primarily the result of BC-driven disruption of their immune system leading to high systemic immunosuppression.

Results herein show that circulating B cells from our cohort of BCPs are different from those of HCs and show several phenotypic, functional and metabolic characteristics associated with increased intrinsic inflammation. In particular, we show that there is a redistribution of B cell subsets, characterized by a decrease in the frequency of naïve B cells and an increase in the frequency of memory B cells, especially DN memory B cell, in the blood of BCPs as compared to HCs, as also previously shown by others (36, 37). Unlike B cells from HCs, B cells from BCPs secrete higher amounts of pro-inflammatory cytokines, which are associated with the activation of the transcription factor NF-kB, and also secrete higher amounts of lactate, associated with hyper-glycolytic B cells. Lactate secretion can be inhibited at least *in vitro* by blocking the lactate transporter that is responsible for signaling through inflammatory pathways. This inflammatory and hyper-metabolic phenotype of B cells in BCPs may represent a significant therapeutic challenge that may account for the reduced immune response to infections and also for the limited efficacy of immunotherapies when combined with chemotherapy.

Our previously published work has identified crucial defects in blood-derived B cells from individuals with systemic low-grade chronic inflammation, inflammaging (38), and associated conditions/diseases, such as aging (21, 39–45), obesity (43), Type-2 Diabetes Mellitus (46–48) and chronic HIV infection (39, 44, 49). B cells from these individuals are characterized by intrinsic inflammation and persistent IA, which contribute to dysfunctional B cells and reduced humoral immunity (50).

The B cell pool of BCPs shows features very similar to the B cell pool of older adults, such as increased memory (in particular DN memory) B cell frequencies, increased secretion of pro-inflammatory cytokines and metabolites, increased expression of nuclear NF-kB, and increased metabolic phenotype. These features suggest that B cells from our cohort of BCPs, prior to having ever received senescence-inducing cancer therapies, such as chemotherapy or radiation, show signs of accelerated aging and cell senescence (not measured in the present study). Accelerated aging is known to promote greater risk for decreased resilience, frailty, cognitive decline, and the onset of age-related chronic disease, for which a similar risk has been found in those diagnosed with cancer (51). Understanding the underlying biological mechanisms through which BC itself and its treatments accelerate biological aging is critical to mitigate the risk of age-related decline in humoral immunity among BCPs, needed to protect this vulnerable population from infectious diseases.

## 5 Limitations

Although results in the present paper show significant B cell differences between our cohorts of BCPs and HCs, a limitation of the study is represented by the small sample size, mainly due to the strict recruitment criteria of BCPs exhibiting similar characteristics and lacking comorbidities. We believe that the homogeneity of BCPs in our cohort may yield a better interpretation of the results while reducing biological confounding factors. It is well known that systemic B cell populations vary significantly depending on BC grade, stage, and patient-specific conditions and comorbidities.

The limited amount of blood drawn from these patients represents another limitation of our study, as mechanistic experiments cannot be done, and therefore establishing cause-and-effect relationships has proven to be challenging with such limited samples. Nonetheless, we believe that the results we obtained with total B cells from these BCPs are invaluable and physiologically relevant, as they are a crucial indicator of the immune status of these patients. Having said that, we agree that our results, although promising, need to be confirmed in larger cohorts of BCPs.

## Data Availability

The original contributions presented in the study are included in the article/**Supplementary Material**. Further inquiries can be directed to the corresponding author.

## In memoriam

This paper is dedicated to the memory of Roland Jurecic, PhD, who passed away in 2025.

## Supplementary material

Supplementary Figure 1 Gating strategies and a representative dot plot from one HC and one BCP. Representative dot blots from one HC (top) and one BCP (bottom). PBMC (2x10^6^/ml) were stained for 20 min at room temperature with Live/Dead kit, and with anti-CD45, anti-CD19, anti-CD27 and anti-IgD fluorochrome-conjugated antibodies to evaluate naive (IgD+CD27-), IgM memory (IgD+CD27+), switched memory (IgD-CD27+), and DN (IgD-CD27-) B cells.

Supplementary Figure 2 Levels of expression of transcripts for glucose transporters in B cells from HCs and BCPs. The mRNA was isolated from unstimulated B cells from HCs and BCPs (among those in **Figure 4A**) and qPCR run to evaluate the expression of transcripts for the glucose transporters Glut2 **(A)**, Glut3 **(B)**, Glut4 **(C)**. Mean comparisons between groups were performed by unpaired Student’s t test (two-tailed). ns=not significant.
